# Resilience-enhancing interventions for antepartum depressive symptoms: systematic review

**DOI:** 10.1192/bjo.2022.60

**Published:** 2022-05-06

**Authors:** Annika L. Walker, Anke B. Witteveen, René H. J. Otten, Corine J. Verhoeven, Jens Henrichs, Ank de Jonge

**Affiliations:** Department of Midwifery Science, AVAG, Amsterdam Public Health Research Institute, Amsterdam UMC, Vrije Universiteit Amsterdam, The Netherlands; Department of Midwifery Science, AVAG, Amsterdam Public Health Research Institute, Amsterdam UMC, Vrije Universiteit Amsterdam, The Netherlands; Medical Library, Vrije Universiteit Amsterdam, The Netherlands; Department of Midwifery Science, AVAG, Amsterdam Public Health Research Institute, Amsterdam UMC, Vrije Universiteit Amsterdam, The Netherlands; Department of Midwifery Science, AVAG, Amsterdam Public Health Research Institute, Amsterdam UMC, Vrije Universiteit Amsterdam, The Netherlands; Department of Midwifery Science, AVAG, Amsterdam Public Health Research Institute, Amsterdam UMC, Vrije Universiteit Amsterdam, The Netherlands

**Keywords:** Psychosocial interventions, perinatal psychiatry, depressive disorders, resilience, antepartum depressive symptoms

## Abstract

**Background:**

Antepartum depressive symptoms (ADS) are highly prevalent and may affect the mother and child. Cognitive–behavioural therapy and interpersonal therapy are effective psychological interventions for depression. However, low adherence and high attrition rates in studies of prevention and treatment of antepartum depression suggest that these approaches might not be entirely suitable for women with mild/moderate ADS. Considering the protective association between resilience and ADS, women with ADS might benefit more from interventions focusing on promotion of mental well-being and resilience.

**Aims:**

We aimed to provide an overview of studies evaluating the effectiveness of antepartum resilience-enhancing interventions targeting the improvement of ante- and postpartum depressive symptoms. We also investigated whether these interventions improve resilience and resilience factors in the peripartum period.

**Method:**

We conducted a systematic review, using PRISMA guidelines. Studies were eligible for inclusion when they utilised a randomised controlled trial or quasi-experimental design, studied pregnant women with ADS, and implemented psychological interventions that (a) aimed to reduce maternal ADS and/or prevent peripartum major depression, and (b) addressed one or more psychological resilience factors.

**Results:**

Five of the six included cognitive–behavioural therapy interventions and all four mindfulness-based interventions were effective in reducing peripartum depressive symptoms and/or the incidence of depression. However, the methodological quality of most of the included studies was low to moderate. Only three studies assessed change in resilience factors.

**Conclusions:**

Resilience-enhancing interventions might be beneficial for mental well-being of pregnant women with ADS, although more rigorously designed intervention studies are needed.

Pregnancy and the transition to motherhood are life-changing experiences for most women, and are accompanied by both physical and psychological changes and challenges that make these women vulnerable to mental health problems.^1,2^ Correspondingly, depressive symptoms during pregnancy are quite prevalent, with up to 20% of women experiencing antepartum depressive symptoms (ADS).^3–5^ Depressive symptoms impair maternal social and physical functioning, and are related to increased maternal distress and poor maternal quality of life.^6–8^ Moreover, if left untreated, ADS can develop into peripartum major depressive disorder, affecting 5–13.5% of women during pregnancy and 7–13.1% in the postpartum period.^4,5,9^ Furthermore, ADS have been shown to be associated with harmful maternal health behaviours and pregnancy complications.^10–12^ Beyond these adverse effects for the woman herself, ADS also affect the health and development of the unborn infant, as maternal ADS are related to preterm birth and low neonatal birth weight.^13–17^ Additionally, offspring exposed to maternal depressive symptoms *in utero* show an increased risk of cognitive, developmental and mental health problems in childhood, adolescence and adulthood.^18,19^ This illustrates that maternal ADS are a serious public health problem, and that early intervention is crucial.

## Interventions for antepartum depressive symptoms

Accordingly, research into the evaluation of the effectiveness of prevention and treatment programmes for antepartum depression has increased. However, evidence regarding the effectiveness of (preventative) interventions for ADS is inconsistent. For major depression,^20^ a meta-analysis found that cognitive–behavioural therapy (CBT) and interpersonal psychotherapy (IPT) are effective treatments. Body-oriented interventions, such as yoga, also seem promising for the treatment of major depression, but because of the limited sample sizes and methodological quality of the included studies, evidence is not conclusive.^20^ Two meta-analyses^21,22^ and a systematic review^23^ that included ADS ranging from women at risk for depression to those with major depressive disorder found that, overall, CBT and IPT are effective interventions. However, effect sizes were larger in more clinical study populations,^21–23^ whereas trials directed at non-clinical populations evaluating the effectiveness of IPT and CBT during pregnancy regularly suffered from low adherence and high attrition rates.^24–27^ Therefore, it could be argued that these treatment approaches might not be entirely suitable for women with mild-to-moderate ADS. Possibly, the nature of these interventions might be too intensive, time-consuming or be experienced as stigmatising.^28–31^ Moreover, (exposure-based) CBT during pregnancy has previously been criticised for its potential adverse (neuro-endocrine) effects.^32^ Hence, in a recent randomised controlled trial, an increase of depressive symptoms in pregnant women receiving CBT and a negative effect of CBT on gestational age at birth was found in anxious women, suggesting CBT to be associated with increased hormonal stress reactions resulting from the confrontational elements of CBT.^32,33^ Accordingly, Lever Taylor et al^34^ proposed that pregnant women with depressive symptoms might benefit more from interventions focusing on the promotion of mental well-being.

## Resilience and antepartum depressive symptoms

This recommendation is consistent with the increasing emphasis on the prevention of mental disorders and the promotion of mental well-being both in general and maternal mental healthcare.^35,36^ One of the key concepts known to be associated with mental well-being is psychological resilience,^37,38^ which is also a known protective factor against the development of depression.^39^ Although the exact definition varies between disciplines, the American Psychological Association defines resilience as ‘the process of adapting well in the face of adversity, trauma, tragedy, threats or significant sources of stress’.^40^ Originally, the concept of resilience has been considered as a stable personality trait that enhances the ability to adapt to adverse experiences.^41^ Yet, recently, resilience is seen as a dynamic and modifiable process that varies across the life course.^37,42^ Consistently, a systematic review of studies in the general population concluded that resilience training based on a combination of mindfulness and cognitive and behavioural skills may be able to improve individual resilience, despite considerable heterogeneity between the trials.^43^ Improved resilience, conceptualised as successful stress recovery, positivity and psychological flexibility, is considered to be preventive in the development and recurrence of depression.^44^ Moreover, a low level of resilience during pregnancy is associated with antepartum and postpartum depression.^45–47^ Third-generation behavioural therapies, such as acceptance and commitment therapy (ACT) and mindfulness-based cognitive therapy, aim to enhance mental well-being by utilising resilience factors, including psychological flexibility and mindfulness.^48,49^

Because of the modifiable nature of resilience, as well as the changes and challenges associated with pregnancy and the transition to motherhood, we expect that training resilience might be a potential beneficial component of interventions for ADS. In the present systematic review, we therefore provide an overview of trials that evaluated the effectiveness of antepartum resilience-enhancing interventions primarily targeting the improvement of mild-to-moderate ante- and postpartum depressive symptoms. We also investigate whether these (psychological) interventions improve resilience and resilience factors in the ante- and postpartum period.

## Method

For this review, we followed the guidelines outlined in the Preferred Reporting Items for Systematic Reviews and Meta-Analyses (PRISMA) statement.^50^ The protocol was registered at the international Prospective Register of Systematic Reviews (PROSPERO; registration number CRD42020123592).

### Search strategy and study selection

A comprehensive search was developed by an experienced information specialist (R.H.J.O.) for the following electronic databases, up to September 2020: PubMed, EMBASE, CINAHL, APA PsycInfo and Cochrane Library. Search terms expressing ‘depression’ were used in combination with search terms comprising ‘pregnant women’ and search terms comprising ‘resilience’, synonyms of resilience or ‘resilience factors’. The full search strategy is included in Supplementary Appendix 1 available at https://doi.org/10.1192/bjo.2022.60.

Two reviewers (A.L.W. and A.B.W.) independently screened all titles and abstracts for eligibility by using Rayyan, a web-based systematic review application (Rayyan Systems Inc., Cambridge, USA; see www.rayyan.ai).^51^ The full texts of potential eligible articles were retrieved and independently reviewed by two authors (A.L.W. and A.B.W.) against eligibility criteria. Disagreements were resolved by discussion and consensus or by consulting a third reviewer (J.H.).

### Eligibility criteria

To be selected for inclusion, studies had to meet the following criteria: be published in a peer-reviewed journal in English, Dutch or German; utilise a randomised controlled trial or quasi-experimental design with one or more control groups; study pregnant women aged ≥18 years with depressive symptoms; and implement an intervention during pregnancy that (a) aimed to reduce maternal ADS and/or prevent the onset of peripartum major depression, measured by validated self-report or standardised structured clinical interviews, and (b) addressed one or more psychological resilience factors. Based on the literature, the following resilience factors were determined *a priori*: cognitive/psychological flexibility, mindfulness, acceptance, optimism or positive attributional style, active coping, self-efficacy and self-esteem.^52–56^

We only considered internal resilience factors and excluded environmental or non-psychological factors, such as social support or physical activity. Studies examining pregnant women at risk for depression (women with prior depression or anxiety) and/or reporting current ADS were included, provided that depression measurements were used during screening. Also, studies examining pregnant women from the general population were included if they reported a subgroup analysis for participants with current depressive symptoms. Studies that did not use measurements of depression or that exclusively studied women with major depression were excluded.

### Quality assessment and data extraction

Methodological quality of the included studies was assessed with the Cochrane Collaboration's tool assessing risk of bias.^57^ Two authors (A.L.W. and A.B.W.) individually assessed the risk of bias of each study and resolved discrepancies through discussion. Each study was assessed at study level as low risk, high risk or unclear risk for selection bias, performance bias, detection bias, attrition bias, reporting bias and ‘other’ bias, resulting in an overall risk of bias appraisal ranging from low to high risk.^57^

Data extraction was performed by A.L.W. and verified by A.B.W. Extracted data included details regarding the design of the trial, participants, the description of the intervention and control condition, outcomes and results (change and difference in means, risk ratio and effect size measures). Because of the heterogeneity of the interventions and the outcome measurements, a narrative synthesis was conducted.

## Results

**Fig. 1:**
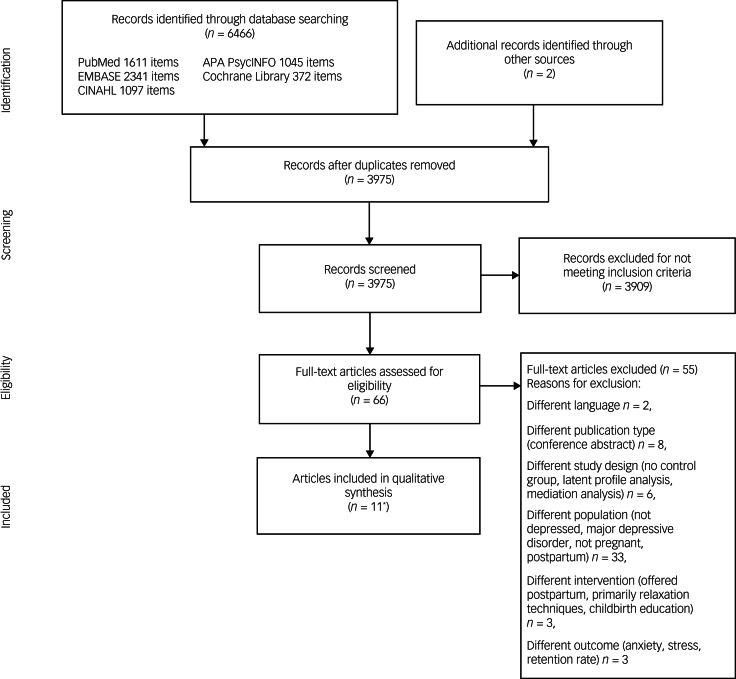
Preferred Reporting Items for Systematic Reviews and Meta-Analyses flow chart. *Of the articles included in the qualitative synthesis, two concerned the same study with different outcome and follow-up measures, which were reported as one study in the present review.

The search yielded a total of 6466 articles. Two additional publications were identified through checking references. After removal of duplicates, titles and abstracts of 3975 articles were screened, resulting in 3909 articles not meeting eligibility criteria and leaving 66 articles for full-text review. Of these, 11 articles were identified as eligible, with two articles reporting different outcome measures of the same trial as reported by Muñoz et al^58^ and Urizar and Muñoz.^59^ Thus, based on the defined inclusion criteria, ten studies were included in the current review. Fig. 1 illustrates the study selection process, using the PRISMA flow diagram.

Table 1 summarises the characteristics of the included studies. Studies were conducted in Western and non-Western countries. Of the ten studies included, nine studies had a randomised controlled study design, and one study^60^ used a pre–post test design with a control group. The interventions employed in the included studies are described in Table 2.

**Table 1:** Characteristics and findings of the included studies

Author (year), country	Study design participants, intervention and control	Eligibility	Intervention and comparator	Outcome measures and assessment time points	Depression severity at baseline	Attrition	Summary of findings (primary outcomes)^a^
Aslami et al (2017),^60^ Iran	Pre–post test with control group *N* = 75 Intervention: 30 (15 high depression, 15 high anxiety Intervention: 30 (15 high depression, 15 high anxiety) Control: 15 (high depression or stress)	BDI-II >14 and/or BAI >7, 16–32 weeks pregnant	Intervention: mindfulness based on Islamic spiritual schemes Intervention: CBT Control: reading condition	BDI-II, BAI T0: 16–32 weeks pregnant T1: post-intervention	BDI-II Intervention: mean 55.6 (s.d. 27.68) CBT: mean 39.6 (s.d. 11.68) Control: mean 52.33 (s.d. 6.91)	Not reported	Significant improvement of depression scores over time of both intervention groups compared with the control group (*P* < 0.001). The mindfulness intervention improved the scores more strongly than CBT (*P* < 0.001)
Fathi-Ashtiani et al (2015),^67^ Iran	RCT *N* = 196 Intervention: 98 Control: 98	BDI ≥13, 18–32 years, uncomplicated singleton pregnancy, literate	Intervention: enhancing cognitive–behavioural skills programme Control: care as usual	BDI, EPDS, BAI, CSEI Religious Attitude Scale Questionnaire, T0: start of the third trimester T1: post-intervention (2 weeks postpartum)	Intervention: BDI mean 21.17 (s.d. 10.92), EPDS mean 17.33 (s.d. 6.87) Control: BDI mean 16.92 (s.d. 4.91), EPDS mean 13.52 (s.d. 5.81)	Intervention: 35% Control: 28%	Significant improvement within the intervention group over time (BDI mean (T1) 14.86 and EPDS mean (T1) 13.05; *P* < 0.001). Controlled for baseline scores, significantly lower BDI and EPDS scores (*P* < 0.05) in the intervention group than in the control group. Significant improvement in self-esteem in both groups over time (intervention mean (T0) 29.09 to mean (T1) 31.81; control mean (T0) 28.74 to mean (T1) 31.12; *P* < 0.001), but not between groups
Guo et al (2020),^66^ China	RCT *N* = 314 Intervention: 157 Control: 157	EPDS ≥9, 18–40 years, second or third trimester until <34 weeks of gestation, Internet access, literate in Chinese	Intervention: Chinese online version of the MBSP programme C: care as usual	EPDS, BDI-II, MAAS, STAI I + II, WHO-5, SCS, PSI, CECPAQ 1-year, IBQ-VSF T0: second or third trimester <34 weeks gestation T1: 3 months postpartum T2: 12 months postpartum	Intervention: EPDS mean 12.5 (s.d. 2.8), BDI-II mean 6.4 (s.d. 3.2) Control: EPDS mean 12.4 (s.d. 2.5), BDI-II mean 6.6 (s.d. 3.8)	Intervention: 8.2% Control: 10.8%	Significant improvement of EPDS scores in the MBSP group from T0 to 3 months (*P* < 0.01) and 12 months (*P* < 0.001) postpartum; EPDS scores in the control group showed no improvement from T0 to 3 months, but significant improvement from T0 to 12 months (*P* < 0.05) postpartum. Compared with the control group, the MBSP group improved significantly more over time (T0 to T2, *P* < 0.01). All participants in the intervention group had EPDS scores <9 at T1 and T2 (mean scores not reported). No results reported for BDI-II. Significant improvement in mindfulness in the intervention group within and between groups over time (*P* < 0.05)
Jesse et al (2015), ^65^ USA	RCT *N* = 146 Intervention: 72 (54 African American) Control: 74 (54 African American) (stratified in low-medium and high risk for depression by EPDS score)	EPDS ≥4 (4–9 low-medium risk; ≥10 high risk), ≥18 years, 6–30 weeks pregnant, rural low-income African American, White and Hispanic women	Intervention: culturally tailored cognitive–behavioural intervention ‘Insight-Plus’ and routine social services Control: care as usual including routine social services	EPDS, BDI-II (questionnaires were read to participants) T0: during pregnancy T1: after the last session T2: 1 month after the intervention	Intervention: low-medium risk EPDS mean 5.42, BDI-II mean 12.25 Control: low-medium risk EPDS mean 5.81, BDI-II mean 10.51 Intervention: high risk EPDS mean 15.19, BDI-II mean 23.48 Control: high risk EPDS mean 14.27, BDI-II mean 23.57. No s.d., only s.e.m. reported	Intervention: 46% Control: 3%	Per-protocol analysis owing to attrition. EPDS and BDI-II scores of the low-risk intervention group, but not the control group, improved over time. In the high-risk groups, EPDS and BDI-II scores of both the intervention and control groups improved significantly from T0 to T1 and T0 to T2: In the low-risk groups, the BDI-II score, but not the EPDS score, improved significantly for the intervention group compared with the control group (mean change 4.92 *v*. 0.59; *P* = 0.018; 5.67 *v*. 1.51, *P* = 0.04). In the high-risk groups, the difference in mean change of both EPDS and BDI-II between intervention and control group was not significant. Subgroup analysis of African American participants only: in the low-risk groups, significant improvement of the BDI-II scores in the intervention group at T1, compared with the control group (mean change 5.20 *v*. 0.70; *P* = 0.02), but not EPDS scores. In the high-risk groups, significant improvement of the EPDS at T1 and T2 in the intervention group compared with the control group (mean change 5.59 *v*. 2.18, *P* = 0.017; 6.32 *v*. 3.14, *P* = 0.037)
Kozinszky et al (2012),^61^ Hungary	RCT *N* = 1762 (general population) Intervention: 728 Control: 1034 Subgroup analysis with APD *n* = 324 Intervention: 119 Control: 205	Pregnant, Leverton Questionnaire score ≥12 for the subgroup	Intervention: preventive group intervention Control: four group meetings with standard information about pregnancy, childbirth and baby care	Questionnaire interview: Leverton Questionnaire, risk factors for depression T0: second trimester T1: 6–8 weeks postpartum	Not reported	Total group Intervention: 2.5% Control: 2.4%	In the subgroup of participants with ADS, 32.8% in the intervention group and 50.7% in the control group reported elevated PPD scores at T1, whereas 67.2% in the intervention group and 49.3% in the control group had no elevated scores, resulting in an absolute risk reduction of 17.8% by the intervention. Women with ADS had significantly higher odds to also have postpartum depressive symptoms compared with women with no ADS, both in the control and intervention groups. Compared with the control group (odds ratio 10.06, 95% CI 7.01–14.42; *P* < 0.001), the odds ratio of PPD for participants with APD in the intervention group (odds ratio 5.01, 95% CI 3.12–8.05; *P* < 0.001) was significantly lower (Mantel–Haenszel test: *P* < 0.001)
Lara et al (2010), ^64^ Mexico	RCT *N* = 377 Intervention: 250 Control: 127	CES-D ≥16 (62.7%) and/or a self-reported history of depression, >26 weeks pregnant, minimum reading ability	Intervention: ‘Salud Mental de Mamás y Bebés/Mothers and Babies Mental Health’ Control: care as usual plus self-help book on depression	Interview: BDI-II, mood disorders on SCID, SCL-90 T0: during pregnancy (>26 weeks) T1: 6 weeks postpartum T2: 4–6 months postpartum	BDI-II ≥14 Intervention: 65.8% Control: 56.4%	Total Intervention: 68.8% Control: 38.6% Between randomisation and first session Intervention: 57.6% Control: 7.8%	Significantly lower cumulative incidence (T1 + T2) of major depression in the intervention group (6/56 = 10.7%) compared with the control group (15/60 = 25%; *P* < 0.05). BDI-II scores improved in both the intervention and the control group over time (time *P* = 0.00 and time×intervention *P* = 0.02), but no significant between-group difference was observed. Controlling for depression at T0, results were similar
Milgrom et al (2011), ^63^ Australia	RCT *N* = 143 High screening score (≥13) Intervention: 21 Control: 22 Low screening score (<13) Intervention: 50 Control: 50	EPDS and/or RAC ≥13, 20–32 weeks pregnant	‘Towards Parenthood’ intervention in addition to community networking Control: care as usual plus information on community networking	BDI-II, DASS-sf, PSI T0: 20–32 weeks gestation T1: 12 weeks postpartum	EPDS Intervention: mean 8.96 (s.d. 5.76) Control: mean 8.75 (s.d. 5.93)	Intervention: 33.8% Control: 41.7%	Significantly less participants at T1 above threshold for depression (BDI-II ≥14) in the intervention compared with the control group (Yates’ corrected χ^2^ = 6.35, *P* < 0.05). Significant improvement in depression scores in the intervention group at T1 compared with the control group, controlling for BDI-II scores at T0 (F_1, 86_ = 7.82, *P* < 0.01; Cohen's *d* = 0.6)
Muñoz et al (2007),^58^ USA	Pilot RCT *N* = 45 Intervention: 21 Control: 20	CES-D ≥16 and/or a major depressive episode in the history, 12–32 weeks pregnant, literate in English or Spanish	Intervention: ‘Mamás y Bebés/Mothers and Babies Course’ Control: care as usual plus information about local social services	CES-D, EPDS, MMS T0: 12–32 weeks of gestation T1: post-intervention T2–T5: 1, 3, 6 and 12 months postpartum	CES-D Intervention: mean 16.00 (s.d. 8.56) Control: mean 16.82 (s.d. 8.05)	8.8%	No significant changes in depression over time within groups. No significant differences between groups in depression scores or incidence of a major depressive episode (intervention 14% *v*. control 25%)
Urizar and Muñoz (2011),^59^ USA	RCT *N* = 57 Intervention: 24 Control: 33	CES-D ≥16 and/or a major depressive episode in the history, 6–28 weeks pregnant, literate in English or Spanish	Intervention: ‘Mamás y Bebés*/*Mothers and Babies Course’ Control: care as usual plus information about local social services	MMS, CES-D, PANAS, VAS for perceived stress, salivary cortisol T0: 6–28 weeks of gestation T1: 6 months postpartum T2: 18 months postpartum	CES-D Intervention: mean 20.6 (s.d. 7.9) Control: mean 23.7 (s.d. 12.3)	T1: 7.8% T2: 13%	Positive affect decreased and negative effect increased over time from T0 to T1 in both groups (*t* = 6.1, *P* < 0.001 and *t* = −6.4, *P* < 0.001), whereas from T1 to T2, positive affect increased and negative effect decreased (*t* = −5.7, *P*< 0.001 and *t* = 6.6, *P* < 0.001) in both groups. No significant between-group differences at T1 nor T2
Yang et al (2019),^62^ China	RCT *N* = 123 Intervention: 62 Control: 61	GAD-7 >4 or PHQ-9 >4, 24–30 weeks pregnant, literate in Chinese Exclusion: GAD-7 >14 or PHQ-9 >14	Intervention: online mindfulness intervention programme Control: care as usual plus psychoeducation and online chat group	PHQ-9, GAD-7, FFMQ T0: 24–30 weeks of gestation T1: post-intervention	PHQ-9 Intervention: mean 5.98 (s.d. 2.24) Control: mean 5.72 (s.d. 2.65)	Intervention: 16.2% Control: 18.1%	Significant improvement of depression in the intervention group, but not in the control group, over time (*t* = 6.218, *P* < 0.001) and between groups (*t* = −5.212, *P* < 0.001). In the intervention group, more participants reported only mild depressive symptoms (PHQ-9 <5) at T1 (intervention 41 *v*. control 22, *P* < 0.003). Significant improvement in mindfulness in the intervention group (*t* = 4.501, *P* < 0.001)
Yazdanimehr et al (2016), ^68^ Iran	RCT *N* = 80 Intervention: 40 Control: 40	EPDS >13 and BAI >16, 6 weeks to 6 months pregnant, at least a high school graduate	Intervention: mindfulness-integrated CBT Control: care as usual	EPDS, BAI T0: during pregnancy T1: post-intervention T2: 4 weeks post intervention	EPDS Intervention: mean 16.83 (s.d. 2.7) Control: mean 16.63 (s.d. 2.64)	Intervention: 17.5% Control: 25%	Significant lower depression scores at T1 and T2 in the intervention group compared with the control (*P* < 0.001)

BDI-II, Beck Depression Inventory-II; BAI, Beck Anxiety Inventory; CBT, cognitive–behavioural therapy; T0, time point 0; T1, time point 1; RCT, randomised controlled trial; BDI, Beck Depression Inventory; EPDS Edinburgh Postnatal Depression Scale; CSEI, Coopersmith Self-Esteem Inventory Adult Form; MBSP, mindfulness-based strengths practice; MAAS, Mindfulness Attention Awareness Scale; STAI I + II, State-Trait Anxiety Inventory I and II; WHO-5, Well-Being Index World Health Organization Five; SCS, Self-Compassion Scale; PSI, Parenting Stress Index; CECPAQ, Comprehensive Parenting Behavior Questionnaire; IBQ-VSF, Infant Behavior Questionnaire-Very Short Form; T2, time point 2; APD, antepartum depression; ADS, antepartum depressive symptoms; PPD, postpartum depression; CES-D, Center for Epidemiologic Studies – Depression Scale; SCID, Structured Clinical Interview; SCL-90, Symptoms Checklist-90; RAC, Risk Assessment Checklist; DASS-sf, Depression Anxiety Stress Scales short form; MMS, Maternal Mood Screener during pregnancy; PANAS, Positive and Negative Affect Schedule; VAS, Visual Analog Scale; GAD-7, Generalised Anxiety Disorder Scale-7; PHQ-9, Patient Health Questionnaire-9; FFMQ, Five Facets of Mindfulness Questionnaire.

a. Effect sizes and *P*-values are provided when reported. Only primary outcomes for depression and resilience or resilience factors are summarised.

**Table 2:** Characteristics of the interventions

Study	Intervention	Modality and length intervention, provider	Resilience component
Aslami et al^60^	Mindfulness based on Islamic spiritual schemes, based on the Mindfulness-based stress reduction protocol by Kabat-Zinn, adapted for pregnancy	Group, 8 weeks with weekly 2 h sessions plus daily homework. Provider not specified	Mindfulness, acceptation
Fathi-Ashtiani et al^67^	Enhancing cognitive–behavioural skills programme, based on CBT, adapted to account for the religious and cultural context of Iran as well as pregnancy	Individually, combined with standard prenatal care visits. Eight sessions, 40–60 min per session plus homework. The programme was provided via recorded videos and an interactive workbook, facilitated by a psychologist	Aims to increase self-esteem
Guo et al^66^	Chinese online version of the mindfulness-based strengths practice programme, focusing on self-compassion. Based on the ‘Mindful self-compassion programme’ by Neff and Germer	Individually, online. Six weeks including six sequential programme steps, with a total of 10 h. Every week, participants performed 6 episodes with guided exercises of 15 min each, with instructions to implement those in daily life and reflect about the exercises in an online diary.	Mindfulness
Jesse et al^65^	Culturally tailored cognitive–behavioural intervention ‘Insight-Plus’, including pregnancy-related psychoeducation and information. Based on ‘Insight’, the cognitive–behavioural model by Beck and the biopsychosocial-spiritual theory by Jesse. Culturally customised to African American, Hispanic and White rural low-income women. Available in English and Spanish	Group, two to six participants. Six weeks, 2 h once a week plus homework and an optional weekly booster session by a staff member (resource mom) via telephone, who also helped with logistic problems related to participation. Participants received an MP3 player with a playlist (guided visualisation, motivational music and self-recorded positive affirmations). Provided by licensed mental healthcare professionals and a clinical social worker	Coping (manage negative thinking, positive self-talk and affirmations)
Kozinszky et al^61^	Preventive group intervention, including psychoeducation about postpartum depression and pregnancy-related content as well as psychotherapy components. Based on elements of IPT and CBT	Group, up to 15 participants per group, plus partners (optionally). Four weeks, starting at 25 weeks gestation, 3 h once a week. Each session consisted of education and exercises and concluded with a relaxation exercise. Provided by psychiatrists and health visitors with training in psychiatry	Development of coping skills, improvement of self-acceptance
Lara et al^64^	‘Salud Mental de Mamás y Bebés/Mothers and Babies Mental Health’, based on a depression prevention programme for non-pregnant women comprising psycho-educational and CBT elements. Includes educational, psychological and group elements, also pregnancy- and postpartum-related information	Group, 5–15 participants. Eight weeks, 2 h a week plus homework. Participants received a workbook. Workbook and sessions included educational and psychological elements and group exercises. Provided by trained facilitators with clinical experience	Aims to increase positive thinking and self-esteem
Milgrom et al^63^	‘Towards Parenthood’ intervention, based on CBT and clinical experience, and focusing on risk factors	Individually, self-help workbook comprising eight modules, seven during pregnancy (one every week) and one at 6 weeks postpartum. Module 2 had to be completed by the partner. The content of each module was discussed via telephone (30 min) with a (trainee) psychologist. Additional community networking	Aims to increase self-esteem, enhance behavioural and cognitive skills for coping with depression and anxiety
Muñoz et al^58^ and Urizar and Muñoz^59^	‘Mamás y Bebés/Mothers and Babies Course’, based on CBT using social learning and attachment theory. Tailored to meet Latino sociocultural needs, available in English and Spanish	Group, three to eight participants. Twelve weeks during pregnancy, weekly sessions with psychoeducational and therapeutical content and stress-management exercises plus homework. Four booster sessions (1, 2, 3 and 12 months postpartum). Provided by clinical psychology doctoral graduate students and postdoctoral fellows, supervised by a licensed clinical psychologist	Self-efficacy and coping skills
Yang et al^62^	Online mindfulness intervention programme, based on mindfulness-based stress reduction therapy and adapted to pregnancy. Utilises mindfulness, attention monitoring, and acceptance theory	Individually, online. Moderated chat function to interact with other participants and researchers. Eight weeks, four sessions of 40 min, plus homework exercises. Each session comprised film, audio and text material about a mindfulness exercise. Participants were contacted by nurses (video/telephone) to discuss problems and experiences with their mindfulness practice. Provided by two nurses, a midwife and supervised by psychologist	Mindfulness, acceptance
Yazdanimehr et al^68^	Mindfulness-integrated CBT, combining CBT and mindfulness.	Group, eight sessions of 90 min. Provided by a trained psychologist (MSc), supervised by a clinical psychologist (PhD)	Mindfulness

CBT, cognitive–behavioural therapy; IPT, interpersonal psychotherapy.

### Methodological quality of the studies

The methodological quality of the studies is illustrated in Table 3. One study was evaluated as having an overall low risk of bias,^61^ whereas the majority of the studies were evaluated with having some concerns^58,59,62–66^ or high risk of bias.^60,67,68^ Except for one study, none of the studies reported blinding procedures. Furthermore, the majority of studies suffered from high and/or unevenly distributed attrition rates. Ratings of ‘unclear’ or ‘high risk of bias’ for the domain ‘Other sources of bias’ were based on baseline imbalance in depression between study groups,^64,67^ no reporting of baseline characteristics^60^ or poor reporting of analyses and study results.^60^

**Table 3:** Quality assessment of included studies as per domain of risk of bias

	Random sequence generation	Allocation concealment	Blinding of participants and personnel	Blinding of outcome assessment	Incomplete outcome data	Selective reporting	Other bias	Overall bias^a^
Aslami, et al^60^	?	x	x	x	?	?	x	x
Fathi-Ashtiani et al^67^	?	?	x	x	x	?	x	x
Guo et al^66^	+	+	x	x	+	x	+	?
Jesse et al^65^	+	?	x	x	x	?	+	?
Kozinszky et al^61^	+	+	+	+	+	?	+	+
Lara et al^64^	+	+	x	x	x	?	x	?
Milgrom et al^63^	+	+	x	x	x	?	+	?
Muñoz et al^58^ and Urizar and Muñoz^59^	+	+	x	x	+	x	+	?
Yang et al^62^	+	+	x	x	+	?	+	?
Yazdanimehr et al^68^	?	?	x	x	x	?	+	x

+ indicates a low risk of bias regarding this domain; ? indicates that the risk of bias is unclear (identification of a potential risk of bias but its influence on the outcome of the study was appraised as unlikely; or insufficient provision of information on methods and procedures); x indicates a high risk of bias regarding this domain.

a. The study was evaluated as having: +, an overall low risk of bias; ?, some concerns regarding the overall risk of bias; or x, an overall high risk of bias.

### Effect of interventions on depressive symptoms

The identified interventions addressing resilience (factors) could be divided into primarily CBT-based and mindfulness-based intervention approaches (see Table 2).

#### CBT-based interventions

Six studies assessed the effect of a CBT-based intervention on peripartum depressive symptoms, with one study focusing on ADS and five focusing on the reduction and prevention of postpartum depressive symptoms. All six interventions were adapted to pregnancy and included psychoeducation about ante- and postpartum depression. Two interventions were offered individually and four in groups consisting of two to fifteen participants (see also Table 2). Five of the studied interventions were at least partly effective in reducing peripartum depressive symptoms and/or the incidence of depression, whereas one study showed no effect (Table 1). However, except for the study by Kozinszky et al,^61^ five of the six studies had a moderate-to-high overall risk of bias.

Kozinszky et al^61^ studied a 4-week preventive group intervention for pregnant women based on psychoeducation and elements of CBT and IPT. The subgroup analysis of women with ADS (*n* = 324) revealed an absolute risk reduction of 17.9% of having elevated postpartum depressive symptoms in women in the group intervention compared with women in the control group following group meetings with standard information about pregnancy. Also, Lara et al^64^ evaluated an 8-week psychoeducational group intervention with components of CBT, combined with group exercises and supported by a workbook, compared with care as usual plus a self-help book on depression (*n* = 377). Depression scores in both groups had improved over time, but the cumulative incidence of major postpartum depression in the intervention group was significantly lower than in the control group. However, the study suffered from a high, unbalanced attrition rate and baseline imbalances for depression (see Table 1 for more detail).

Milgrom et al^63^ examined the effect of a CBT-based self-help workbook with eight weekly modules and support sessions via telephone, during pregnancy and one time at 6 weeks postpartum, in women at risk for depression and with elevated ADS (*n* = 143). At 12 weeks postpartum, depressive symptoms in the intervention group had strongly improved (Cohens *d* = 0.6) and fewer participants scored above the cut-off for depression, compared with the control group that had received usual care. Fathi-Ashtiani et al^67^ (*n* = 196) also developed an eight-session CBT programme provided individually, using a workbook and video material adapted to the Iranian religious and cultural context. Controlled for baseline scores, post-intervention depressive symptoms in the intervention group were significantly lower than in the control group; however, the results of this study were assessed as highly likely to be biased. Jesse et al^65^ evaluated a 6-week culturally tailored group CBT intervention for 146 low-income African American, Hispanic and White women in the USA. Participants were stratified into low, medium and high risk, based on their initial depression score. Depressive symptoms (Beck Depression Inventory-II) in the low-risk group had improved post-intervention compared with the control group, whereas no treatment effect was found in the high-risk group. In the subsample of African American women, the high-risk CBT group showed improved depression scores (Edinburgh Postnatal Depression Scale) compared with the high-risk control group. The intervention was thus found to be effective for some of these subgroups. However, the unevenly distributed attrition rate and the small sample size of the subgroups may have influenced these results. Finally, a 12-week CBT group intervention that was culturally tailored to pregnant Latino women with depressive symptoms and/or a past major depressive episode^58,59^ showed no differences between groups on depression scores, incidence of major depression, and positive and negative affect, measured at different follow-up time points (*n* = 45 and *n* = 57).

#### Mindfulness-based interventions

Four studies evaluated the effect of mindfulness-based interventions, of which three aimed to decrease ADS^60,62,68^ and one aimed to prevent postpartum depressive symptoms up to 12 month after birth.^66^ Three interventions targeted women with anxiety and (mild) depressive symptoms.^60,62,68^ Two interventions were provided in groups^60,68^ and two were provided individually online.^62,66^ All four mindfulness-based interventions improved depressive symptoms significantly compared with their control conditions. However, there were some concerns regarding the overall risk of bias in two studies,^62,66^ and two studies had a high risk of bias,^60,68^ which might have led to an overestimation of the effects.

An individual programme using the approach of mindfulness-based strengths practice significantly improved depressive symptoms (Edinburgh Postnatal Depression Scale score only) at 3 and 12 months postpartum, compared with usual care (*n* = 314).^66^ The 6-week programme included guided exercises focused on self-compassion and was provided online. A second individual online intervention, based on mindfulness-based stress reduction, including an interactive chat function and telephone contact, was effective in reducing post-intervention depression scores (*n* = 123).^62^ The 8-week intervention consisted of four sessions plus homework, and was compared with a control group participating in online psychoeducation and a chat group.

Yazdanimehr et al^68^ evaluated mindfulness-integrated CBT in group sessions, compared with care as usual. The 8-week intervention significantly improved post-intervention ADS, although the study was rated as having a high risk of bias (Table 3). Moreover, Aslami et al^60^ reported a significantly higher reduction of post-intervention depressive symptoms for women participating in an 8-week group intervention applying mindfulness and Islamic spiritual schemes, compared with both CBT and usual care. However, this study used a quasi-experimental design and risk of overall bias was rated as high (see Table 3).

### Effect of interventions on resilience (factors)

None of the studies meeting the inclusion criteria addressed the improvement of resilience directly. Identified resilience components addressed in the selected studies comprised mindfulness, acceptance, coping (positive thinking and self-talk) and self-esteem. Only three of the studies measured change in these resilience factors across the pre- and post-intervention period. Both online mindfulness interventions (mindfulness-based stress reduction and mindfulness-based strengths practice) improved mindfulness scores significantly compared with the control condition post-intervention^62^ and at 3 and 12 months postpartum.^66^ In the study examining a CBT programme adapted to the Iranian religious and cultural context, self-esteem had increased in both groups from baseline, at the start of the third trimester, to 2-week postpartum assessments, showing no effect favouring the intervention.^67^

## Discussion

To our knowledge, this is the first systematic review providing an overview of studies evaluating the effectiveness of antepartum resilience-enhancing interventions that aim to reduce ante- and postpartum depressive symptoms and prevent peripartum depression among pregnant women with mild-to-moderate depressive symptoms. According to our criteria, we included ten studies, of which five CBT-based and four mainly mindfulness-based interventions were at least partly effective in reducing peripartum depressive symptoms and/or the incidence of postpartum depression. However, the methodological quality of most of the included studies was low to moderate, which might have led to an overestimation of effects. Only one of the included studies demonstrating the effectiveness of a preventive group intervention based on a combination of psychoeducation and elements of CBT and IPT was rated of high methodological quality.^61^ The CBT group intervention adapted to Latina culture evaluated by Muñoz et al^58^ and Urizar and Muñoz^59^ was the only non-effective intervention, yet the sample size was small and the study might have been underpowered to detect a significant difference. Identified resilience factors addressed by the interventions were mindfulness, acceptance, coping (including positive thinking and self-talk) and self-esteem. However, only three studies assessed change in these factors, and the construct of resilience itself was not directly addressed.

Although earlier reviews have revealed a more mixed efficacy of antepartum interventions based on CBT or mindfulness,^21,34^ interventions addressing resilience and resilience factors might be promising in improving peripartum depressive symptoms as revealed by the present systematic review. In contrast to these previous reviews, we included only studies aimed at secondary prevention. This may partly explain the relative consistent pattern of results of the studies included in this review. Moreover, considering the earlier reported negative association between ADS and resilience,^46,47^ the findings of the current review indeed support our hypothesis that interventions increasing resilience may be especially beneficial for women with low-to-moderate symptoms, and may secondarily prevent the development of peripartum clinical depression. Our results and earlier literature on antepartum CBT and IPT interventions suggest that psychological approaches, such as classical CBT and IPT, might be more effective for the treatment of clinical peripartum depression,^21–23^ whereas pregnant women with mild-to-moderate depressive symptoms might benefit more from resilience-enhancing interventions.^28,32^ Both CBT- and mindfulness-based intervention approaches addressing resilience factors seem promising. However, these findings should be replicated in methodologically rigorous trials.

Attrition rates in four of the CBT-based studies^63–65,67^ were high or differential, potentially leading to bias. This has previously been described as a methodological problem in trials examining peripartum psychological interventions.^34,69^ In three of the mindfulness-based interventions attrition was low, and occurred slightly more frequently in the control group. Two of these interventions were provided as online programmes, which is in line with low attrition rates reported in previous research on online interventions in the peripartum period.^70^ All selected interventions, except for the mindfulness-integrated CBT evaluated by Yazdanimehr et al,^68^ were adapted or specifically designed for expectant mothers and included psychoeducational elements on pregnancy, ante- and postpartum depressive symptoms and motherhood. This might enhance engagement and contribute to the effectiveness of these interventions.

The second aim of the present study was to investigate whether the identified psychological interventions improve resilience and resilience factors in the ante- and postpartum period. Interestingly, none of the included studies directly addressed the construct of resilience. However, components of resilience that were addressed by the interventions included mindfulness, acceptance, coping (including positive thinking and self-talk) and self-esteem. Nevertheless, change in these factors across the pre- and post-intervention period was assessed in only three studies. Both online mindfulness-based interventions significantly increased mindfulness post-intervention^62^ and postpartum.^66^ Fathi-Ashtiani et al,^67^ who evaluated the effectiveness of an adapted and culturally-specific mindfulness programme for the Iranian context, observed that the increase in self-esteem did not differ between the intervention group and care as usual. To shed more light on effect mechanisms involved, future research should include process measures alongside primary outcomes.

In addition to mindfulness- and CBT-based interventions, we also expected to identify third-generation behavioural therapies such as ACT for the prevention and treatment of peripartum depression, as ACT is increasingly popular and comprises resilience factors, including psychological flexibility and mindfulness.^48,49^ A recent meta-analysis demonstrated the effectiveness of ACT in successfully reducing mild depressive symptoms in the general population.^71^ Bonacquisti et al^72^ developed a rationale for an antepartum four-session ACT intervention, suggesting that the emphasis of ACT on the enhancement of psychological flexibility instead of an emphasis on the reduction of depressive symptoms may lead to higher mental well-being. This might be especially beneficial for pregnant women, as it may reduce feelings of (self-)stigmatisation, and positively influence somatic complaints and the adjustments related to the transition to motherhood.^30,72^ However, our search identified only one non-eligible study (because of inadequate study population characteristics) showing that ACT had improved quality of life and anxiety in pregnant Iranian women,^73^ as well as a pilot study without a control group, which observed that an antepartum ACT group intervention was feasible and had improved both psychological flexibility and depressive symptoms. In addition, we found two ongoing trials evaluating the impact of ACT on depressive symptoms and anxiety.^74,75^ In line with a recent review of reviews of psychological interventions for peripartum depression,^76^ we conclude that ACT seems promising, but more research assessing its impact on ADS is needed.

### Strengths and limitations

This study has several strengths. As far as we are aware, it is the first review of resilience-enhancing psychological interventions during pregnancy. A comprehensive search was conducted, followed by systematic screening, quality assessment and review of the studies performed independently by two researchers. Nevertheless, there are a few limitations. We only included studies that were peer-reviewed and published in English, Dutch or German, which might have biased our results. Furthermore, although we included randomised controlled trials or trials using a quasi-experimental design with control group, the conclusions of the present review are limited because the methodological quality of the individual studies was rated as low to moderate, with the exception of one high-quality study. Moreover, as only two studies reported effect sizes, assumptions about the clinical significance regarding the effectiveness of the successful interventions are difficult to make. Our conclusions also might be influenced by the limited number of studies included. However, we included studies with interventions aimed at pregnant women with elevated depressive symptoms and/or risk factors (women with prior depression or anxiety) only, as implementation of these interventions in obstetric mental healthcare is suggested to be more feasible than primary prevention interventions. The second reason for the limited number of studies was that we only included interventions provided during pregnancy, as early treatment of ADS is essential regarding the adverse effects on the pregnant woman and her unborn child.^77,78^ Finally, we were only able to conduct a narrative synthesis based on the studies included in the current systematic review, as a meta-analysis was not considered feasible because of the heterogeneity of the interventions.

In conclusion, our results suggest that antepartum psychological interventions addressing the enhancement of resilience factors, such as mindfulness, acceptance, coping and self-esteem, seem effective in improving peripartum depressive symptoms. The ten interventions identified could be divided into primarily CBT-based and mindfulness-based intervention approaches. However, the methodological quality of the included studies was mostly low to moderate, which must be considered when interpreting the results. In contrast to our expectations, no interventions using an ACT approach were included. Considering the adverse effects of peripartum depression on the mental and physical well-being of mothers and their (unborn) children, the promotion of well-being and prevention of exacerbation of depressive symptoms during pregnancy are essential. Therefore, future research should invest in more rigorously designed studies evaluating the effectiveness of antepartum resilience-enhancing interventions, using appropriate process measures, and should report measures of effect to enable future meta-analyses. Moreover, future studies should particularly investigate the role of resilience in ACT interventions for the reduction of ADS, to improve the mental well-being of pregnant women and their children.

## Data Availability

Data availability is not applicable to this article as no new data were created or analysed in this study.

## Supplementary material

For supplementary material accompanying this paper visit http://doi.org/10.1192/bjo.2022.60. click here to view supplementary material
