# Supplementary material for: Resilience-enhancing interventions for antepartum depressive symptoms: systematic review
Source: BJPsych Open. 2022 May 6;8(3):e89. doi: 10.1192/bjo.2022.60 (PMC9169502; doi:10.1192/bjo.2022.60)
Supplement: Supplementary file 1 [file S2056472422000606sup001.docx]

**Search strategy in PubMed (2020 September 9^th^)**

| **#** | **Query** | **Results** |
| --- | --- | --- |
| **#4** | #1 AND #2 AND #3 | **1,611** |
| **#3** | ("Resilience, Psychological"[Mesh] OR "Adaptation, Psychological"[Mesh:noexp] OR "Emotional Adjustment"[Mesh] OR "Sense of Coherence"[Mesh] OR "Self Concept"[Mesh:noexp] OR "Self Efficacy"[Mesh] OR "Mindfulness"[Mesh] OR "Optimism"[Mesh] OR "Hope"[Mesh] OR resilien*[tiab] OR "locus of control"[tiab] OR "sense of coherence"[tiab] OR adaptive behavior[tiab] OR adaptive behaviour[tiab] OR cognitive flexibility[tiab] OR coherence sense[tiab] OR coping behavior[tiab] OR coping behaviour[tiab] OR coping skill*[tiab] OR emotional adaptation*[tiab] OR emotional adjustment*[tiab] OR mindful*[tiab] OR optimism*[tiab] OR positive thinking[tiab] OR psychologic adaptation[tiab] OR psychologic adjustment*[tiab] OR psychologic flexibility[tiab] OR psychological adaptation[tiab] OR psychological adjustment*[tiab] OR psychological flexibility[tiab] OR self-concept[tiab] OR self-confidence[tiab] OR self-efficacy[tiab] OR self-esteem*[tiab] OR self-perception*[tiab] OR hardiness[tiab] OR hope[tiab]) | **304,069** |
| **#2** | ("Depression"[Mesh] OR "Depressive Disorder"[Mesh:noexp] OR "Depression, Postpartum"[Mesh] OR "Depressive Disorder, Major"[Mesh] OR "Dysthymic Disorder"[Mesh] OR depress*[tiab] OR dysthymi*[tiab]) | **507,830** |
| **#1** | ("Pregnancy"[Mesh] OR "Pregnant Women"[Mesh] OR "Perinatal Care"[Mesh] OR "Prenatal Care"[Mesh] OR pregnan*[tiab] OR antenatal[tiab] OR prenatal[tiab] OR perinatal[tiab] OR ante natal[tiab] OR pre natal[tiab] OR peri natal[tiab] OR antepartum[tiab] OR ante partum[tiab] OR peripartum[tiab] OR peri partum[tiab] OR antepartal[tiab] OR ante partal[tiab] OR peripartal[tiab] OR peri partal[tiab]) | **1,074,682** |

**Search strategy in Embase.com (2020 September 9^th^)**

| **#** | **Query** | **Results** |
| --- | --- | --- |
| **#4** | #1 AND #2 AND #3 | **2341** |
| **#3** | 'psychological resilience'/exp OR 'resilience'/exp OR 'coping behavior'/de OR 'meaning-making'/exp OR 'stress management'/exp OR 'psychological adjustment'/de OR 'self concept'/exp OR 'self esteem'/exp OR 'sense of coherence'/exp OR 'mindfulness'/exp OR 'optimism'/exp OR 'hope'/exp OR 'hardiness'/exp OR resilien*:ti,ab,kw OR 'locus of control':ti,ab,kw OR 'sense of coherence':ti,ab,kw OR 'adaptive behavior':ti,ab,kw OR 'adaptive behaviour':ti,ab,kw OR 'cognitive flexibility':ti,ab,kw OR 'coherence sense':ti,ab,kw OR 'coping behavior':ti,ab,kw OR 'coping behaviour':ti,ab,kw OR 'coping skill*':ti,ab,kw OR 'emotional adaptation*':ti,ab,kw OR 'emotional adjustment*':ti,ab,kw OR mindful*:ti,ab,kw OR optimism*:ti,ab,kw OR 'positive thinking':ti,ab,kw OR 'psychologic adaptation':ti,ab,kw OR 'psychologic adjustment*':ti,ab,kw OR 'psychologic flexibility':ti,ab,kw OR 'psychological adaptation':ti,ab,kw OR 'psychological adjustment*':ti,ab,kw OR 'psychological flexibility':ti,ab,kw OR 'self-concept':ti,ab,kw OR 'self-confidence':ti,ab,kw OR 'self-efficacy':ti,ab,kw OR 'self-esteem*':ti,ab,kw OR 'self-perception*':ti,ab,kw OR hardiness:ti,ab,kw OR hope:ti,ab,kw | **426516** |
| **#2** | 'depression'/de OR 'dysthymia'/exp OR 'major depression'/exp OR 'minor depression'/exp OR 'perinatal depression'/exp OR depress*:ti,ab,kw OR dysthymi*:ti,ab,kw | **763446** |
| **#1** | 'pregnancy'/exp OR 'pregnant woman'/exp OR 'perinatal care'/exp OR 'perinatal period'/exp OR 'prenatal care'/exp OR 'prenatal period'/exp OR pregnan*:ti,ab,kw OR antenatal:ti,ab,kw OR prenatal:ti,ab,kw OR perinatal:ti,ab,kw OR 'ante natal':ti,ab,kw OR 'pre natal':ti,ab,kw OR 'peri natal':ti,ab,kw OR antepartum:ti,ab,kw OR 'ante partum':ti,ab,kw OR peripartum:ti,ab,kw OR 'peri partum':ti,ab,kw OR antepartal:ti,ab,kw OR 'ante partal':ti,ab,kw OR peripartal:ti,ab,kw OR 'peri partal':ti,ab,kw | **1201999** |

**Search strategy in CINAHL (via EBSCO ; 2020 September 9^th^)**

| **#** | **Query** | **Results** |
| --- | --- | --- |
| **S4** | S1 AND S2 AND S3 | **1,097** |
| **S3** | (MH "Hardiness") OR (MH "Adaptation, Psychological") OR (MH "Coping") OR (MH "Self Concept") OR (MH "Self-Efficacy") OR (MH "Mindfulness") OR (MH "Optimism") OR (MH "Hope") OR TI (resilien* OR (locus W1 control) OR (sense W1 coherence) OR (adaptive W0 behavior) OR (adaptive W0 behaviour) OR (cognitive W0 flexibility) OR (coherence W0 sense) OR (coping W0 behavior) OR (coping W0 behaviour) OR (coping W0 skill*) OR (emotional W0 adaptation*) OR (emotional W0 adjustment*) OR mindful* OR optimism* OR (positive W0 thinking) OR (psychologic W0 adaptation) OR (psychologic W0 adjustment*) OR (psychologic W0 flexibility) OR (psychological W0 adaptation) OR (psychological W0 adjustment*) OR (psychological W0 flexibility) OR (self W0 concept) OR (self W0 confidence) OR (self W0 efficacy) OR (self W0 esteem*) OR (self W0 perception*) OR hardiness OR hope) OR AB (resilien* OR (locus W1 control) OR (sense W1 coherence) OR (adaptive W0 behavior) OR (adaptive W0 behaviour) OR (cognitive W0 flexibility) OR (coherence W0 sense) OR (coping W0 behavior) OR (coping W0 behaviour) OR (coping W0 skill*) OR (emotional W0 adaptation*) OR (emotional W0 adjustment*) OR mindful* OR optimism* OR (positive W0 thinking) OR (psychologic W0 adaptation) OR (psychologic W0 adjustment*) OR (psychologic W0 flexibility) OR (psychological W0 adaptation) OR (psychological W0 adjustment*) OR (psychological W0 flexibility) OR (self W0 concept) OR (self W0 confidence) OR (self W0 efficacy) OR (self W0 esteem*) OR (self W0 perception*) OR hardiness OR hope) OR SU (resilien* OR (locus W1 control) OR (sense W1 coherence) OR (adaptive W0 behavior) OR (adaptive W0 behaviour) OR (cognitive W0 flexibility) OR (coherence W0 sense) OR (coping W0 behavior) OR (coping W0 behaviour) OR (coping W0 skill*) OR (emotional W0 adaptation*) OR (emotional W0 adjustment*) OR mindful* OR optimism* OR (positive W0 thinking) OR (psychologic W0 adaptation) OR (psychologic W0 adjustment*) OR (psychologic W0 flexibility) OR (psychological W0 adaptation) OR (psychological W0 adjustment*) OR (psychological W0 flexibility) OR (self W0 concept) OR (self W0 confidence) OR (self W0 efficacy) OR (self W0 esteem*) OR (self W0 perception*) OR hardiness OR hope) | **177,880** |
| **S2** | (MH "Depression") OR (MH "Depression, Postpartum") OR (MH "Dysthymic Disorder") OR TI (depress* OR dysthymi*) OR AB (depress* OR dysthymi*) OR SU (depress* OR dysthymi*) | **184,119** |
| **S1** | (MH "Pregnancy") OR (MH "Expectant Mothers") OR (MH "Perinatal Care") OR (MH "Prenatal Care") OR TI (pregnan* OR antenatal OR prenatal OR perinatal OR (ante W0 natal) OR (pre W0 natal) OR (peri W0 natal) OR antepartum OR (ante W0 partum) OR peripartum OR (peri W0 partum) OR antepartal OR (ante W0 partal) OR peripartal OR (peri W0 partal)) OR AB (pregnan* OR antenatal OR prenatal OR perinatal OR (ante W0 natal) OR (pre W0 natal) OR (peri W0 natal) OR antepartum OR (ante W0 partum) OR peripartum OR (peri W0 partum) OR antepartal OR (ante W0 partal) OR peripartal OR (peri W0 partal)) OR SU (pregnan* OR antenatal OR prenatal OR perinatal OR (ante W0 natal) OR (pre W0 natal) OR (peri W0 natal) OR antepartum OR (ante W0 partum) OR peripartum OR (peri W0 partum) OR antepartal OR (ante W0 partal) OR peripartal OR (peri W0 partal)) | **264,523** |

**Search strategy in APA PsycInfo (via EBSCO ; 2020 September 9^th^)**

| **#** | **Query** | **Results** |
| --- | --- | --- |
| **S4** | S1 AND S2 AND S3 | **1,045** |
| **S3** | DE "Adaptability (Personality)" OR DE "Psychological Endurance" OR DE "Resilience (Psychological)" OR DE "Emotional Control" OR DE "Emotional Adjustment" OR DE "Coping Behavior" OR DE "Sense of Coherence" OR DE "Self-Concept" OR DE "Self-Esteem" OR DE "Mindfulness" OR DE "Optimism" OR DE "Hope" OR TI (resilien* OR (locus W1 control) OR (sense W1 coherence) OR (adaptive W0 behavior) OR (adaptive W0 behaviour) OR (cognitive W0 flexibility) OR (coherence W0 sense) OR (coping W0 behavior) OR (coping W0 behaviour) OR (coping W0 skill*) OR (emotional W0 adaptation*) OR (emotional W0 adjustment*) OR mindful* OR optimism* OR (positive W0 thinking) OR (psychologic W0 adaptation) OR (psychologic W0 adjustment*) OR (psychologic W0 flexibility) OR (psychological W0 adaptation) OR (psychological W0 adjustment*) OR (psychological W0 flexibility) OR (self W0 concept) OR (self W0 confidence) OR (self W0 efficacy) OR (self W0 esteem*) OR (self W0 perception*) OR hardiness OR hope) OR AB (resilien* OR (locus W1 control) OR (sense W1 coherence) OR (adaptive W0 behavior) OR (adaptive W0 behaviour) OR (cognitive W0 flexibility) OR (coherence W0 sense) OR (coping W0 behavior) OR (coping W0 behaviour) OR (coping W0 skill*) OR (emotional W0 adaptation*) OR (emotional W0 adjustment*) OR mindful* OR optimism* OR (positive W0 thinking) OR (psychologic W0 adaptation) OR (psychologic W0 adjustment*) OR (psychologic W0 flexibility) OR (psychological W0 adaptation) OR (psychological W0 adjustment*) OR (psychological W0 flexibility) OR (self W0 concept) OR (self W0 confidence) OR (self W0 efficacy) OR (self W0 esteem*) OR (self W0 perception*) OR hardiness OR hope) OR KW (resilien* OR (locus W1 control) OR (sense W1 coherence) OR (adaptive W0 behavior) OR (adaptive W0 behaviour) OR (cognitive W0 flexibility) OR (coherence W0 sense) OR (coping W0 behavior) OR (coping W0 behaviour) OR (coping W0 skill*) OR (emotional W0 adaptation*) OR (emotional W0 adjustment*) OR mindful* OR optimism* OR (positive W0 thinking) OR (psychologic W0 adaptation) OR (psychologic W0 adjustment*) OR (psychologic W0 flexibility) OR (psychological W0 adaptation) OR (psychological W0 adjustment*) OR (psychological W0 flexibility) OR (self W0 concept) OR (self W0 confidence) OR (self W0 efficacy) OR (self W0 esteem*) OR (self W0 perception*) OR hardiness OR hope) | **325,426** |
| **S2** | DE "Major Depression" OR DE "Dysthymic Disorder" OR DE "Postpartum Depression" OR DE "Recurrent Depression" OR DE "Depression (Emotion)" OR TI (depress* OR dysthymi*) OR AB (depress* OR dysthymi*) OR KW (depress* OR dysthymi*) | **312,896** |
| **S1** | DE "Pregnancy" OR DE "Perinatal Period" OR DE "Prenatal Care" OR TI (pregnan* OR antenatal OR prenatal OR perinatal OR (ante W0 natal) OR (pre W0 natal) OR (peri W0 natal) OR antepartum OR (ante W0 partum) OR peripartum OR (peri W0 partum) OR antepartal OR (ante W0 partal) OR peripartal OR (peri W0 partal)) OR AB (pregnan* OR antenatal OR prenatal OR perinatal OR (ante W0 natal) OR (pre W0 natal) OR (peri W0 natal) OR antepartum OR (ante W0 partum) OR peripartum OR (peri W0 partum) OR antepartal OR (ante W0 partal) OR peripartal OR (peri W0 partal)) OR KW (pregnan* OR antenatal OR prenatal OR perinatal OR (ante W0 natal) OR (pre W0 natal) OR (peri W0 natal) OR antepartum OR (ante W0 partum) OR peripartum OR (peri W0 partum) OR antepartal OR (ante W0 partal) OR peripartal OR (peri W0 partal)) | **77,898** |

**Search strategy in the Cochrane Library (via Wiley ; 2020 September 9^th^)**

| **#** | **Query** | **Results** |
| --- | --- | --- |
| **#4** | #1 and #2 and #3 | **372** |
| **#3** | resilien*:ti,ab,kw OR (locus NEAR/2 control):ti,ab,kw OR (sense NEAR/2 coherence):ti,ab,kw OR (adaptive NEXT behavior):ti,ab,kw OR (adaptive NEXT behaviour):ti,ab,kw OR (cognitive NEXT flexibility):ti,ab,kw OR (coherence NEXT sense):ti,ab,kw OR (coping NEXT behavior):ti,ab,kw OR (coping NEXT behaviour):ti,ab,kw OR (coping NEXT skill*):ti,ab,kw OR (emotional NEXT adaptation*):ti,ab,kw OR (emotional NEXT adjustment*):ti,ab,kw OR (mindful*):ti,ab,kw OR (optimism*):ti,ab,kw OR (positive NEXT thinking):ti,ab,kw OR (psychologic NEXT adaptation):ti,ab,kw OR (psychologic NEXT adjustment*):ti,ab,kw OR (psychologic NEXT flexibility):ti,ab,kw OR (psychological NEXT adaptation):ti,ab,kw OR (psychological NEXT adjustment*):ti,ab,kw OR (psychological NEXT flexibility):ti,ab,kw OR (self NEXT concept):ti,ab,kw OR (self NEXT confidence):ti,ab,kw OR (self NEXT efficacy):ti,ab,kw OR (self NEXT esteem*):ti,ab,kw OR (self NEXT perception*):ti,ab,kw OR (hardiness):ti,ab,kw OR (hope):ti,ab,kw | **33628** |
| **#2** | depress*:ti,ab,kw OR dysthymi*:ti,ab,kw | **84650** |
| **#1** | pregnan*:ti,ab,kw OR antenatal:ti,ab,kw OR prenatal:ti,ab,kw OR perinatal:ti,ab,kw OR “ante natal”:ti,ab,kw OR “pre natal”:ti,ab,kw OR “peri natal”:ti,ab,kw OR antepartum:ti,ab,kw OR “ante partum”:ti,ab,kw OR peripartum:ti,ab,kw OR “peri partum”:ti,ab,kw OR antepartal:ti,ab,kw OR “ante partal”:ti,ab,kw OR peripartal:ti,ab,kw OR “peri partal”:ti,ab,kw | **66916** |
